# Progressive Training for Motor Imagery Brain-Computer Interfaces Using Gamification and Virtual Reality Embodiment

**DOI:** 10.3389/fnhum.2019.00329

**Published:** 2019-09-26

**Authors:** Filip Škola, Simona Tinková, Fotis Liarokapis

**Affiliations:** Faculty of Informatics, Masaryk University, Brno, Czechia

**Keywords:** brain-computer interface, motor imagery, embodiment, body ownership transfer, gamification

## Abstract

This paper presents a gamified motor imagery brain-computer interface (MI-BCI) training in immersive virtual reality. The aim of the proposed training method is to increase engagement, attention, and motivation in co-adaptive event-driven MI-BCI training. This was achieved using gamification, progressive increase of the training pace, and virtual reality design reinforcing body ownership transfer (embodiment) into the avatar. From the 20 healthy participants performing 6 runs of 2-class MI-BCI training (left/right hand), 19 were trained for a basic level of MI-BCI operation, with average peak accuracy in the session = 75.84%. This confirms the proposed training method succeeded in improvement of the MI-BCI skills; moreover, participants were leaving the session in high positive affect. Although the performance was not directly correlated to the degree of embodiment, subjective magnitude of the body ownership transfer illusion correlated with the ability to modulate the sensorimotor rhythm.

## 1. Introduction

Research in brain-computer interfaces (BCIs) promises to provide humans with the possibility to interact with arbitrary tools using direct connection to the brain. In practice, communication and control of robots or computer systems is the aim of BCI research. BCIs are unique tools for the reason that they are the first ones allowing people to manifest their will without any kind of movement required in the process (Wolpaw et al., 2002), allowing severely paralyzed persons to communicate with their surroundings (Kübler et al., 2004). While an ideal BCI would allow communication using arbitrary conscious activity, so far only a few direct brain communication paradigms have been invented. This study is focused on the BCI paradigm that exploits changes in ongoing neural activity during periods of imagined bodily movement, commonly known as the motor imagery (MI). The control strategy of MI-BCIs requires the users to consciously imagine performing a bodily movement. This process has pronounced electroencephalographic (EEG) neural correlates, mostly consisting of lateralized event-related desynchronization (ERD) of mu (8–12 Hz) and beta (15–30 Hz) neural rhythms over the motor cortex parts corresponding to the imagined body part moving (Pfurtscheller and Lopes da Silva, 1999; Pfurtscheller and Neuper, 2001).

To accurately use MI-BCIs, users must produce stable ERDs, achieved by modulation of sensorimotor rhythm (SMR). Although most people have some SMR modulation ability (Dickhaus et al., 2009), training is usually needed to achieve reasonable accuracy in control of the MI-BCI. During the training, ERD patterns in the user are strengthened (Kaiser et al., 2014), and machine learning processes the signals in the BCI system (Lotte et al., 2015). The human participant trains with the help of neurofeedback, while sophisticated feature extraction and classification algorithms are used in the BCI system side in this non-trivial co-adaptation process (Lotte et al., 2015).

One of the many problems that prevents BCIs from practical adoption is the performance variation among population and the closely associated BCI-illiteracy phenomenon, showing that an estimated 15–30% of the population cannot develop the ability to control BCI systems based on mental imagery or event-related potentials, such as the P300 wave (Dickhaus et al., 2009). On the other hand, there is evidence against BCI-illiteracy, at least in the steady-state visual evoked potentials (SSVEP), which work more robustly and after only very short training (Guger et al., 2012).

Most of the BCI research has been focused on advances in the realms of signal processing, feature extraction, and classification (Chavarriaga et al., 2017). Nevertheless, a trend in the recent BCI research highlights the importance of the human-facing side of the brain interface (Lotte et al., 2013; Jeunet et al., 2016a,b; Sollfrank et al., 2016; Kosmyna and Lécuyer, 2017). The standard training protocols, guiding users through the obligatory training process, were especially criticized for their sub-optimality, often ignoring elementary recommendations about training from psychology, e.g., having tasks in progressive or adaptive fashion, or leveraging of a rich, multi-modal feedback (see Jeunet et al., 2016b for details). It has been hypothesized that even the BCI-illiteracy issue may be caused by improperly designed training, rather than being rooted in physiological origin (Jeunet et al., 2016a).

Commonly used training (based on the Graz protocol, Pfurtscheller and Neuper, 2001) for MI-BCIs is externally paced, event-driven, and makes use of symbolic guidance. The user performs MI in successive trials (usually 30–40 in one run) while the feedback is represented as an extending bar, proportional to the classifier confidence on the current trial. It is not uncommon that study participants cannot make sense of the feedback or find it difficult to focus on the process of MI and the feedback at the same time (Jeunet et al., 2016a; Škola and Liarokapis, 2018).

This study is focused on two following problems with the traditional MI-BCI training. The first issue is split attention (Sweller et al., 1998), emerging when participants performing the imagery need to comprehend their performance and progress using symbolic guidance in visual modality. We hypothesize that freeing the participant from this kind of distraction and transferring the feedback into an easily integrable channel will help the participants to engage the imagery and to easily accommodate them to the training. The second issue this study deals with is the form of the traditional training, which has been identified as unengaging and possibly demotivating. With the current form of the training, participants must have strong intrinsic motivation to adhere to the procedure, or they might be easily discouraged by its simplistic and heavily repetitive form.

We tackle these issues from two sides; firstly, we leverage virtual embodiment, property of immersive virtual reality (VR) with realistic avatar (Kilteni et al., 2012), and deliver training feedback using the participant's surrogate virtual body. BCI feedback mapped onto new “owned” body parts using body ownership transfer has been previously identified as beneficial for more efficient MI-BCI training (Braun et al., 2016; Penaloza et al., 2018; Škola and Liarokapis, 2018). Secondly, we reimplemented the training procedure using gamification techniques. Gamification aims to improve the immersion and motivation in non-entertainment fields using implementation of features from games (de Freitas, 2011). Our gamified training makes use of themed environment and score points, while the challenge arises mainly from the progressive increase in speed across several training runs, or levels (categories of motivational affordances were adapted from Hamari et al., 2014).

Our VR training environment is set in outer space, participants were transferred inside a cockpit of a virtual starship, and their task was to shoot asteroids approaching the nearby Earth-like planet using MI of left and right hands. Feedback was mediated visually using avatar movements in VR and using vibrations delivered to the corresponding hand. Additionally, score was displayed after each training trial and after the entire training run, to provide participants with accurate quantification of their performance. Training runs were presented with increasing pace to keep participants motivated and to increase the information transfer rate in the span of the experimental session. Motivation and mood of the participants were assessed before the experiment, and affect was assessed after the training. Our results (average peak accuracy in the session = 75.84%) indicate the proposed training environment improves MI-BCI operation performance in a naïve and non-experienced population. Moreover, participants were in high positive affect after the training. We also provide additional evidence that subjective degree of embodiment in VR during embodied training positively correlates with SMR modulation abilities.

## 2. Background

### 2.1. Action-Driven Body Ownership Illusions

Sense of ownership (SoO) toward a body denotes the subjective experience of owning a body (Gallagher, 2000). Self-attribution of own body is essential for normal human existence and rarely questioned concept by healthy population. However, body ownership illusions using targeted sensory manipulation can temporarily override perceived bodily image and cause partial or full body ownership transfer (first described in the rubber hand illusion, created by Botvinick and Cohen, 1998). Contrary to earlier hypotheses presuming the key role of visual dominance in the rubber hand illusion, it is now widely accepted (Ehrsson et al., 2005; Ehrsson, 2012) that the rubber hand illusion, similarly to other body ownership illusions, emerges due to manipulated multisensory integration processes. Multisensory integration is one of the main mechanisms behind the process of bodily self-attribution, mediated by continuous monitoring of inputs from available sensory modalities (visual, proprioceptive, tactile, etc.), and their simultaneous processing to plausible image of the bodily self (Ehrsson, 2012).

Of special interest for the self-recognition is the process comparing a person's intentions to expected sensory outcomes (Tsakiris et al., 2005), known in the literature as “central monitoring theory” of action recognition or the “comparator model” (David et al., 2008). According to this model, an initiated voluntary action is accompanied by its efference copy. For motor actions, this means that when bodily movement occurs, it is compared to the efference copy of the intent, if such intent existed. In case of match, the observed action is self-attributed, while in the case of afferent sensory signaling not preceded by a corresponding motor command, the observed action is attributed to an external cause (von Holst and Mittelstaedt, 1950; Jeannerod, 2007).

Self-identification (in terms of SoO) of the acting body in action self-attribution is then proceeded under normal circumstances. The experience of having motor control and the subjective experience of controlling one's own actions and intentions, termed the sense of agency (SoA) (Blanke and Metzinger, 2009), is normally bound to the bodily SoO (although SoO and SoA can be dissociated as demonstrated by Kalckert and Ehrsson, 2012).

SoA is often described in the context of control of motor actions; however, the SoA can be treated as a more general concept that represents feeling of authorship of intent in the brain, covering also covert actions such as the capability to create a thought in the stream of thoughts (Gallagher, 2000, 2007). For the action recognition process, one must feel the SoA toward one's own actions, which can be disturbed in neurological or psychiatric illnesses, e.g., schizophrenia (with symptoms such as thought insertion or delusions of control, Gallagher, 2000).

While the rubber hand illusion is based on manipulation of visual and tactile inputs, illusory transfer of the SoO can be mediated by illusions based on voluntary actions—more specifically, by exploiting congruency between the efferent signals conveying motor commands and the re-afferent signals (visual and proprioceptive feedback) (Dummer et al., 2009). In VR, full or partial body transfer illusion occurs when users experience control of their virtual avatars, usually by means of body tracking (Slater et al., 2009).

In the context of the VR, illusory effect of transfer of the SoO, SoA, and sense of bodily self-location is termed virtual embodiment (or just embodiment). Kilteni et al. (2012) define embodiment as follows: “Sense of embodiment toward a body B is the sense that emerges when B's properties are processed as if they were the properties of one's own biological body.”

### 2.2. Agency for BCI Actions and Embodied BCI Training

Experiencing a high SoA is critical during the process of gaining control of a tool—the degree of agency toward it influences the attitude toward the technology, and the resulting performance (Vlek et al., 2014). Eliciting the SoA in the strictly non-motor BCI context is not a straightforward task, as users are facing a new challenge and must rely solely on the synthetic feedback provided by the implementation of the BCI system. Evans et al. (2015) explored the SoA for MI-BCI actions (on a trial-by-trial basis), finding that agency is decreased with introduction of a delay or other discrepancy between the action and the feedback (this reduces the SoA in the traditional bodily context as well). This was however not true for the cases when the visual discrepancy corrected a poorly controlled BCI action. The authors hypothesize that as an only re-afferent feedback on the BCI action, the visual feedback is what the feeling of the SoA is based on during BCI control.

As described in section 2.1, SoA for bodily actions of virtual avatar is the essential principle behind virtual embodiment. If the congruent “bodily feedback” suffices for the illusory transfer of the SoO, could the same kind of feedback (mapped onto movements of a virtual avatar), but synchronized with the MI instead of movement, help to convey the SoA for BCI actions by creating the sense of embodiment?

Vourvopoulos et al. (2016) created an embodied MI-BCI training environment based on the Graz training protocol. The training events were complemented with VR feedback based on motor observation from the first-person perspective. The study further presents an embodied game for MI training based on motor priming. The game consists of a rowing simulator and has self-paced progress, thus it serves to train the user, rather than gradual co-adaptation between the machine and the human participant, as is common in the event-based MI-BCI training paradigms. More recent research (Vourvopoulos et al., 2019) demonstrated the beneficial effect of EEG-based embodied neurofeedback to patients after stroke. The benefits of neurofeedback were most prominent in participants with severe motor impairments. In their preprint, Juliano et al. (2019) studied the specific effect of embodiment onto the MI-BCI control performance in a VR study controlled using the standard training protocol. The study is limited by using 12 participants and only one class of MI (imagined right hand movement), but the results show consistent effect of spatial embodiment positively influencing the BCI performance in VR condition.

Besides the VR context, embodiment is an important topic in robot control, as it can facilitate telepresence. The possibility to control a humanoid robot to carry out complex tasks such as fetching objects using BCIs was demonstrated in Bell et al. (2008). Martens et al. (2012) aimed at embodied robot control using combination of event-related potentials and SSVEP control strategies for an EEG-based BCI and with visual feedback in HMD. The authors suggest that synchronization of a robot's movements with the user's representation of his or her own movements could to lead to increased embodiment. Still, SSVEP is often used in BCI-mediated robotic control, mostly due to the higher accuracy. The low bandwidth issue of current BCI systems is compensated with the usage of semi-autonomic approaches leveraging localization, visual object recognition and targeted control (Petit et al., 2015). Another experiment with SSVEP-controlled robot was done by Kishore et al. (2014), who implemented two control strategies for the robot: SSVEP and eye tracking. Both strategies allowed a strong body ownership transfer illusion, but SSVEP control lead to stronger SoO than eye tracking.

MI paradigm for embodied control of robots using fMRI was prototyped in study by Cohen et al. (2012), who effectively mediated BCI control of a robot located in a different country thousands of kilometers away from the fMRI chamber. Experiments done by Alimardani et al. (2013, 2016b) succeeded in eliciting the SoO toward a humanoid robot controlled by MI-BCI. SoA aroused during MI-BCI manipulation with robotic hands or avatars in VR has been demonstrated to facilitate binding of the body ownership transfer (Perez-Marcos et al., 2009; Braun et al., 2016; Alimardani et al., 2018). Alimardani et al. (2016a) reported that in the span of one session, there was not a significant learning difference between embodied MI-BCI control using human-like hands or a pair of metallic grippers. However, the group training MI skills using humanoid hands performed better in a follow-up session, suggesting a positive role of humanoid embodiment for BCI control.

Using the same humanoid robot as in previous studies by Penaloza et al. (2018) conducted a study focused on the traditional event-based MI training using the robot's movements as the visual feedback. Participants trained using the embodied feedback performed better in the evaluation task than control group participants trained using standard Graz training. Both groups received the visual stimulation using LCD display but still maintained some level of illusory body ownership transfer.

Those results are consistent with our previous study (Škola and Liarokapis, 2018), comparing Graz training using visualization on a standard computer screen to an embodied VR training. A similar methodology, equipment, and set-up were used in the last study; experimental group participants received real-time VR feedback using hand movements of the virtual avatar, but the training lasted only 2 runs and the 3rd run used for evaluation was shorter (10 trials per class). Control group training consisted of standard Graz training without VR. Embodied training led to better BCI performance and a trend was apparent between the magnitude of embodiment and ability to modulate the SMR, but it was not significant. Stronger SMR modulation was also present in the experimental group, but the effect was not significant due to very high variance in performance (both in terms of ERD strength and control accuracy).

### 2.3. Motivation, Attention, and Their Influence to the BCI Performance

To study the role of motivation on BCI performance, metrics used previously in MI-BCI research by Nijboer et al. (2008, 2010) were employed. They quantified the motivation in four dimensions according to Vollmeyer and Rheinberg (1998): mastery confidence (participants' belief in successful mastering of the BCI training task), incompetence fear (level of anxiety connected to anticipated failure in the task), challenge, and interest. In their work, the level of MI-BCI control with visual feedback correlated positively to mastery confidence and the mood in the healthy participants, whereas it was negatively correlated to the incompetence fear (Nijboer et al., 2008). Previous experiments by Leeb et al. (2007) also confirmed a strong role of motivation to performance in the MI-BCI task (navigation in a virtual apartment). The improvement of underperforming subjects was especially significant when the motivation was stronger (by changing the task environment from the standard display to VR). Nevertheless, motivation was examined only qualitatively in this experiment.

Publication by Jeunet et al. (2016b) provides a systematic investigation into the psychological and cognitive factors influencing MI-BCI performance. Cognitive states identified as predictors of BCI performance were motivation (Nijboer et al., 2008; Hammer et al., 2012), mood (Nijboer et al., 2008), and attention (Grosse-Wentrup and Schölkopf, 2012).

A problem commonly occurring in MI-BCI training stems from the need to split attention during training with the standard protocol. This effect is apparent when “two or more sources of attention must be processed simultaneously in order to derive meaning from material” (Sweller et al., 1998). We argue that this is the case when participants are required to visualize bodily movement while being presented with symbolic visual feedback at the same time. Presenting the feedback in the embodied, “bodily domain” should alleviate the split attention issue. While the VR environment can be seen as more visually cluttered compared to standard symbolic feedback on a display (and thus leading to even larger issues with focusing attention), the virtual bodily representation should be processed as if it is one's own body after the body transfer illusion is aroused (Kilteni et al., 2012). The comparator model engaged during action self-recognition is handled by subconscious processes, and it is not impaired in environment with rich visual stimuli. We believe that when the observed bodily actions reflect manifestation of corresponding imagery and complement it in the expected way, one does not require as much additional cognitive processing as in the case with symbolic feedback. To further compensate deficiencies in the non-visual sensory domain, we opted to accompany the embodied visual feedback with vibrotactile stimulation of the corresponding hand.

## 3. Materials and Methods

### 3.1. Participants

Twenty four participants were recruited for the experiment. Out of these, 5 participants were excluded from the experiment for the following reasons: two participants failed to adhere to the instructions (too many hand movements were produced), one participant did not surpass chance level during the first four runs of the experiment, and two participants were excluded because of technical failure during the experimental session (controller vibration malfunction in one case, and severely damaged EEG recording in the other case). Data from the remaining 19 participants (7 females) were used for analysis; their median age was 26 (min. 21, max. 30, *SD* = 2.780). This study was approved and carried out in accordance with the recommendations of the local ethics committee of Masaryk University. All subjects gave written informed consent in accordance with the Declaration of Helsinki.

Participant recruitment was done using an ad in a university magazine article and by contacting participants from our previous experiment (all participants were contacted, regardless of their results) (Škola and Liarokapis, 2018). Consequently, some of the participants have had previous experience with a BCI. Specifically, 9 participants experienced one MI-BCI training session more than a year ago, and one participant experienced three MI-BCI sessions, one in the last year. However, statistical testing confirmed that the previous experience with BCI did not affect the results (for details, see section 4.6.1). Participants received no material compensation for taking part in the experiment.

### 3.2. Apparatus

**Figure 1:**
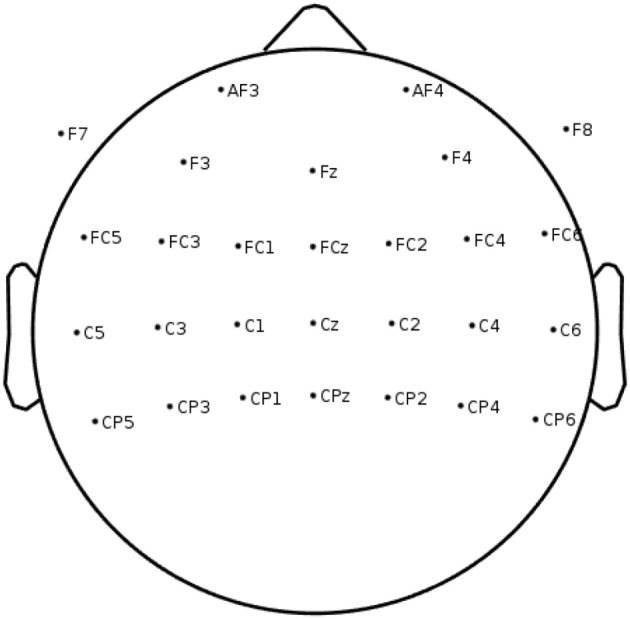
EEG channel locations used in the on-line BCI feedback loop and for the off-line analysis.

EEG data were recorded using a wireless lightweight EEG system Neuroelectrics Enobio 32^1^ with 28 electrodes centered around the motor cortex (see Figure 1 for the details on electrode set-up). Electrodes (AgCl NG Geltrode) were placed using a neoprene cap, following the standard 10–10 system for high-resolution EEG recording. Common mode sense/driven right leg (CMS/DRL) earclip served as a reference for the signals.

**Figure 2:**
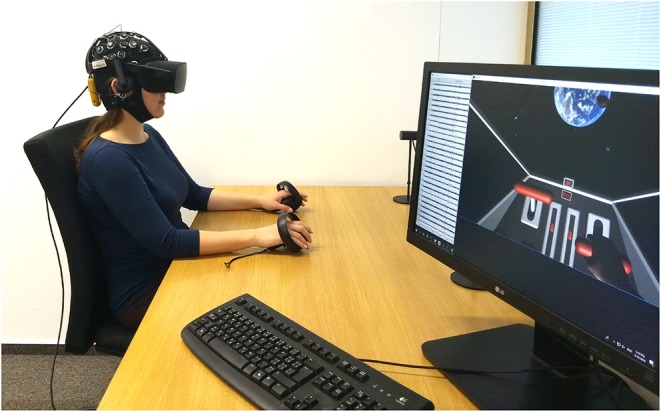
Participant with EEG and VR HMD during the training process finishing a right hand MI trial.

To immerse participants into the VR environment, participants wore a state-of-the-art HMD Oculus Rift CV1^2^ (resolution 1,080 × 1,200 per eye, 90 Hz refresh rate, 110° field of view, rotational and positional tracking). Rift HMD was chosen due to its suitability together with EEG recording (as was confirmed by our previous studies) and its anatomically shaped Oculus Touch controllers. The Touch controllers provided vibrotactile feedback and monitored participants' hand movements. Participants did not actively interact with the controllers, they served only as support for the hands during the training runs. A participant during the experiment is displayed in Figure 2.

Thanks to the mobile EEG system used, Rift HMD did not have to be mounted using special equipment. Participants were equipped with the EEG cap and after ensuring satisfactory signal quality, HMD was positioned with experimenter's help. Signal quality was re-checked again after HMD was put on.

### 3.3. Experimental Design

This study was focused on co-adaptive MI-BCI training using gamified tasks and progressive pace. All participants took part in the same procedure, and all training runs were set in the same VR environment. The first run consisted of MI facilitated by motor observation in VR. The second to fifth runs provided participants with real-time embodied feedback and had progressively faster pace. The last run was dedicated to an experiment inquiring into modality change (post-trial discrete feedback instead of real-time feedback).

The training task took the form of a game of destroying asteroids using left/right hand MI, while the implementation of the underlying training protocol was loosely based on the Graz protocol. Participant performance was evaluated in terms of trial-wise accuracy (i.e., percentage of trials where participant maintained the correct mental state for the majority [>50%] of its duration). This approach to evaluation was motivated by practical use of a BCI for issuing discrete commands using the MI.

To leverage the properties of embodied VR, participants were instructed to synchronize their MI with the avatar movements. At the same time, exploring mental strategies was encouraged, but within the boundaries of avatar movement. Feedback was provided using three modalities; (1) movements of the avatar, (2) vibrations, (3) providing information about trial accuracy (score).

Firstly, the avatar was programmed to adjust the speed of its hand movements according to the classifier decisions on participant's actions. Movements were slowed down when the classifier did not recognize the current state of a participant as the one specified by the current training trial, and slower avatar movements in turn urged the participant to find a more suitable MI strategy. On the other hand, producing recognizable MI patterns led to restoration of the avatar movement speed to the baseline level. In any case, the avatar animations were initiated only after the participant switched to the required MI state first.

Secondly, the controllers were set to vibrate during the periods of a recognized correct state. Thirdly, as a complementary feedback, participants received information about trial accuracy after its completion and after the entire run was finished. Participants received an instruction to maximize the number of trials with a score of 50 or more.

Runs 2–5, although consisting of the same task, were performed with progressively increasing speed. This was achieved by shortening the total time for a trial and speeding up the animation (exact values regarding the animation speed in different runs are provided in section 3.7). As the participants' task was to maintain the correct MI state proportionally to the trial duration, less MI time was needed to finish the trial successfully, but at the same time, participants were required to enter the MI state faster after instruction was displayed.

The last run of the experiment was designed to inquire whether removal of the real-time feedback significantly impairs the performance. Participants were again asked to imagine lateralized hand movement, but the avatar-mediated and vibrotactile feedback modalities were not provided. Results of each trial were shown after end of the MI period (the avatar moved accordingly and the score was displayed, but vibrotactile feedback was not provided). Again, participants had to maintain the MI state for at least 50% of time in order to trigger the BCI command.

### 3.4. Training Application

Application providing the training had a gamified form aiming at increasing levels of attention and motivation during the training procedure. The gamification was accomplished using a themed virtual scene with human-like avatar and game-like progression of difficulty levels. The application for training was developed using the Unity game engine^3^, version 2018.2.14f1.

**Figure 3:**
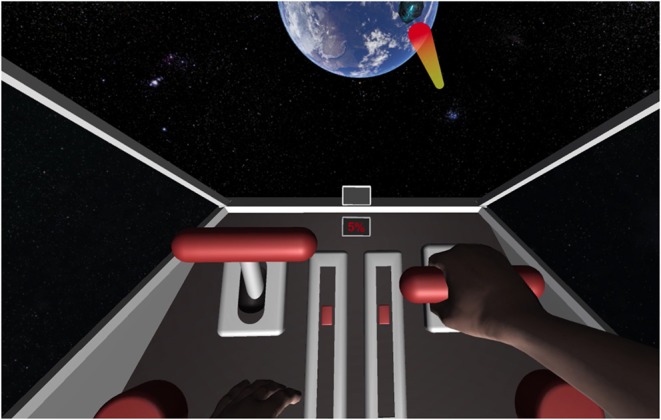
VR view on a successful end of right hand MI trial.

The VR scene was set in outer space and the participant was virtually transferred inside a cockpit of a spaceship with an Earth-like planet ahead. The spaceship contained a simplistic control panel consisting of a low number of interaction elements that triggered weapons aiming at asteroids flying toward the Earth-like planet (see Figure 3). The goal of a training trial was to shoot flying asteroids using MI of the left or right hand, depending on its source position (flying from the left or the right side of the spaceship). The asteroid was destroyed in case the participant was able to produce recognizable MI for at least 50% of the total trial time. In case the trial performance was bellow 50%, the asteroid was destroyed after a timeout (not using the spaceship weapons).

Length of each trial was determined by the run number and participant performance in the trial; therefore, even though it was event driven, the training was not externally paced. Better performance was rewarded by shortening of the training run (but maximal duration of trials was limited). To prevent habituation of the procedure, rest phases consisted of a constant and a random component.

Two virtual displays were placed near the center of the field of view in VR: progress display and instruction display. While the former was used solely to provide information about temporal progression of the current run (using percentage of completed trials), the latter display showed instruction/status concerning the current trial. More specifically, instruction was either start eye fixation (“+” sign), start of the left MI (left arrow sign), start of the right MI (right arrow sign), invalid trial (explained later in this section), or start of the rest period (blank display or score displayed). During the runs with feedback, the instruction display showed the score immediately after the end of each trial (thus displaying score indicated start of the rest period).

Design of VR applications used concurrently to EEG recording is subject to many limitations. Firstly, VR encourages bodily interaction; secondly, VR encourages visual exploration of the scene. Both of these properties of virtual environments would contribute to producing bodily movements that in turn produce electromyographical artifacts disturbing recording of the encephalic origins. To reduce bodily movements, participants were placed into the VR environment for pretraining first, to become familiar with the VR environment (by active exploration of the scene) before the actual EEG recording phase started.

Hand movements produced during the training runs were detected using the VR controllers. In case one of the controllers moved during the ongoing trial, the trial was invalidated and the participant was informed about the detected movement by means of the instruction display (three “x” symbol). Generation of eye movements was suppressed using the eye fixation element on the instruction display (participants were specifically instructed to not follow the flying asteroid with their gaze, but to rather perceive the rest of the scene using peripheral vision; by fixating their eyes to the instruction display, participants could see both the virtual hand movements and the asteroids being destroyed).

The animations were created using keyframing on a freely available rigged humanoid avatar model (downloaded from Mixamo^4^), and the rest of the scene (spaceship, interaction elements) was modeled manually. The avatar's head was deleted from the scene so the participants did not see the internals of the model during set-up. The used avatar represented a male, but its hands had an androgynous look (see Figure 3), thus they were not replaced for female participants. To keep participants in a natural resting position, they had a fully customizable swivel chair available, and the VR camera was adjusted to an anatomically plausible position.

EEG data recording and classification was handled by Openvibe (Renard et al., 2010). Unity and Openvibe were connected directly using a local TCP socket (both applications run on the same computer). Our experimental application provided events using a connection to Openvibe Acquisition Server, and classification results were gathered using connection to Openvibe Designer running the BCI scenario.

### 3.5. Procedure

The experimental session was opened by providing participants with detailed informed consent containing information about the study. This introductory document consisted of two parts; firstly, participants were briefly familiarized with the concept of BCI, MI-BCIs, and motivation to study BCIs (one page). Secondly, the following information about the experimental session were provided: duration of the runs, limitations during EEG recording, and instructions to perform kinesthetic MI (one page). The purpose of the document was not only to prepare the participants for the experimental session, but also to raise the intrinsic motivation by providing meaningful information about the purpose of the study and by ensuring participants that their time spent in the session was valuable, regardless of their performance (this was explicitly stated in the document).

A pre-experiment questionnaire surveying each participant's mood and motivation followed. After filling in the questionnaire, the experimenter explained further details about the experiment. Specifically, objectives of the gamified VR training and the role of symbolic instructions in VR were provided. Participants were seated in the VR tracking area and the HMD was handed out. Its customization was explained (interocular distance setting) and, after HMD was put on, the viewpoint of the virtual environment was set up.

In pre-training, the experimental application was launched in demo mode. Participants had the chance to freely explore the VR scene, and most importantly, they were asked to prepare for imagery of the left and right hand movements. For that purpose, the demo mode in the experimental application consisted of an exact simulation of the first run of the training procedure, with the avatar performing left/right hand MI in randomized order. Time for pre-training was not limited; however, no participant pre-trained for the duration of the entire 40 trials in one run.

Set-up of the EEG device took approximately 30 min. After ensuring satisfactory signal quality, HMD was carefully positioned on the participant, and the signal quality was re-checked. Before the start of the first run, instructions regarding bodily movements were repeated (with special emphasis put on special cases possibly creating muscle artifact contamination or resembling motor action execution, such as eyelid movement during periods of intense imagery or muscle tension).

Six runs of MI-BCI training were then performed. After finishing each run, the experimenter trained the classifier (1–2 min) and the next run started immediately after. Participants rested during the breaks, and the HMD was not taken off if this was not specifically requested by the participant. Participants were virtually present in the Oculus home environment during the break with HMD on. Before the 6th run of the experiment with feedback modality change, participants were shown the altered progression of trials (feedback after the end of a trial—avatar hitting the spaceship buttons, no vibrations provided).

After the last run, the experimenter helped the participants to take off the HMD and the EEG cap and handed out the post-experiment questionnaires (surveying embodiment and affect, and qualitative questionnaire). The average total training time was approximately 28.5 min.

### 3.6. On-Line Feature Construction

Raw EEG data were processed into the features for classifier training and subsequent on-line classification during the training runs with feedback (runs 2–6). The whole procedure was carried out in Openvibe. Data were first digitally filtered using Butterworth passband filter with order 5 in range 8–30 Hz. Spatial filtering was performed using common spatial patterns (CSP) with 3 filters for each class, and epochs were generated using sliding windows with a length of 1 s each 1/16th of a second.

Features were extracted from the epoched signal by applying the Hamming Window and Fast Fourier Transform (FFT). Using FFT, 4-dimensional features were generated using the band power in the following bands: 8–12, 12–16, 16–20, and 20–30 Hz. After logarithmic transformation, these features were passed to a linear discriminant analysis (LDA) classifier with regularized covariance matrix (and automatically generated shrinkage coefficient) for training.

Onset of each MI trial was recognized using markers in the EEG data, as received from the experimental application. As trial lengths varied between the runs in a session, length of the generated epochs for training were as follows: epochs from runs 1–2 were 4 s long, epochs from run 3 were 3.5 s long, epochs from run 4 were 3 s long, and epochs from run 5 were 2.5 s long. Classifier for run 2 was trained with EEG signals from run 1, classifier for run 3 was trained with signals from run 2, classifier for run 4 was trained with concatenated signals from runs 2 and 3, classifier for run 5 was trained with concatenated signals from runs 3 and 4, and classifier for run 6 was trained with concatenated signals from runs 4 and 5. CSP re-calibration took place before each classifier re-training.

### 3.7. Feedback Generation

During runs 2–5, classification results were gathered each 1/16th of a second to adjust the feedback in the training application. To adjust the speed of animations naturally, changes were applied gradually. After initial experimentation, it was decided to set the step for changing the speed to ±1/4 of the last speed value (changes were applied each 1/16th of a second). Speed limits were 20–100% of the initial speed in the run (lower boundary was set to prevent incompetence frustration).

In practice, if a participant performed poorly in a trial, it took approximately 350 ms of incorrect mental state to drop to 20% animation speed from the initial speed. In such cases, it took 250 ms of correct MI state to increase the speed from bottom limit to 48.828% of the speed and another 250 ms to reach the full speed.

After the trial was successfully finished or timeout occurred, the rest of the animations was played using initial speed. Animations in runs 1 and 2 had the same speed. After that, run 3 speed = 2x run 1 speed, run 4 speed = 3x run 1 speed, and run 5 and 6 speed = 4x run 1 speed.

Score feedback aimed to provide the participants with precise quantification of success rate after each trial. Only values between 1 and 100 were displayed; in case the participant spent all trial time in the other MI state, zero was not displayed and the display was kept blank. In the end of each run, participants were presented with the total score, i.e., sum of scores over all trials in the current run. Participants could use this information to compare their performances across the runs.

Vibrotactile feedback provided participants information about the classification result. The controller of the active hand was set to vibrate lightly during correct classification in trials. Strength of the vibrations was adjusted for each participant during the calibration run, as some of the participants claimed to not feel the vibrations at all, while some participants found the vibrations too strong and disturbing.

To quantify latency of the feedback generation mechanism, latencies of each component are considered. EEG data were acquired with 500 samples per second, and drift correction with a threshold of 2 ms was applied. The specification of latency of the used VR system is not officially available, however testing using older generation of the HMD demonstrated 45 ms (Raaen and Kjellmo, 2015) or 46 ms latency time (Oculus, 2013), while unofficial information^5^ on motion-to-photon latency of the used HMD claims it to be <5 ms. Consequently, the majority of delay between the participant and system is created during the epoching. Epochs were created using a sliding window each 1/16th of a second, meaning that feedback was generated based on 62.5-ms-old data. Total latency of the feedback generation mechanism is less than 100 ms, i.e., small enough to not compromise perceived SoA.

### 3.8. EEG Data Processing for Off-Line Analysis

Cleaning, processing and analysis of the EEG data was performed via EEGLAB (Delorme and Makeig, 2004). Due to considerable amounts of noise generated during the EEG recording concurrent to HMD usage in a non-shielded room, we first re-sampled the signals to 100 Hz (to cut off 50 Hz line noise; our frequency range of interest ended at 30 Hz) and high-pass filtered at 1.5 Hz. Cleaning was done using automatic subspace reconstruction (Chang et al., 2018, available as plugin for EEGLAB) and independent component analysis (ICA). Bad channels rejected with automatic subspace reconstruction were interpolated using the spherical interpolation model in EEGLAB. Epochs for ICA (and following analysis steps) were generated from 0.75 s pre-stimulus to 4 s post-stimulus (stimulus was the instruction to start left/right hand MI). ICA decomposition was calculated for each dataset separately, and artifactual components were identified using the Multiple Artifact Rejection Algorithm (Winkler et al., 2014).

ERSPs were computed for each dataset from the following EEG channels: C4, CP4, C2, FC4, and C6 for the left hand; and C3, CP3, C1, FC3, and C5 for the right hand. From the ERSP courses, we further calculated event-related desynchronization (ERD) in the frequency range 8-30 Hz. ERDs were averaged over the duration of the MI epoch according to the run number (4, 3.5, 3, or 2.5 s) for both hands and used as representations of SMR modulation abilities.

Classification accuracy per dataset was gathered using Openvibe trainer using the same settings as in on-line feature construction (details are provided in section 3.6).

We leveraged the EEG signals to compute an index of fatigue levels in each run of the experiment. Spectral analysis was performed on the latter half of the trials, and the fatigue index was computed from the ratio of theta/alpha power spectral density (adapted from Cao et al., 2014).

### 3.9. Questionnaires

A pre-experiment questionnaire surveyed the participants' motivation in the following dimensions: interest, mastery confidence, incompetence fear and challenge (Vollmeyer and Rheinberg, 1998). The questionnaire consisted of 18 questions, based on the questionnaire in Nijboer et al. (2008), translated into Czech. The post-experiment questionnaire surveyed the participants' magnitude of embodiment and affect. The embodiment questionnaire was based on the original rubber hand illusion questionnaire (Botvinick and Cohen, 1998) and psychometric questionnaire for embodiment created by Longo et al. (2008). All questions were answered on a 7-point Likert scale. The English version of both questionnaires can be found in the Supplementary Materials.

Embodiment was surveyed on the following subscales: SoO, SoA, illusory loss of the hands (feelings of not being able to move one's own hands, or of one's own hands disappearing), and illusion of being located at the position of the virtual body. The last variable was adapted from the rubber hand illusion questionnaires, where the position of the rubber hand necessarily differs from the actual position of the participant's hand. In this experiment, this variable denotes susceptibility of the participants to feel the position of their hands to be driven by the animations (i.e., lifting from a table and moving spaceship controls).

## 4. Results

### 4.1. On-Line Accuracy

Accuracy was calculated as the percent of targets hit in a run (target was hit if classifier decision overlapped with the training task for the majority of time in the trial). Bit transfer rate (BTR) was calculated using accuracy and length of a trial in each run. BTR, contrary to accuracy, reflects the increasing pace of the training runs. The formula from Shannon (1949) was used (the original formula is taking into account variable trial length, as used in Krausz et al., 2003):

(1) $$\begin{array}{r} BTR=\left[log_{2}N+P*log_{2}P+\left(1-P\right)*log_{2}\frac{1-P}{N-1}\right]\frac{60}{trial\;length} \end{array}$$

Where N (number of classes) equals 2, and probability P corresponds to a participant's accuracy in a run. Trial length is the actual average length of trial in a run, including the rest period.

The average of the best accuracy achieved during the session was 75.84% (min = 60%, max = 92%, SD = 11.251). Peak performance was gained most frequently in the 3rd run, with its average accuracy = 67.11%. This is an improvement from our previous study (Škola and Liarokapis, 2018) where the experimental group (*N* = 15) gained an average hit-wise accuracy of 63.33% (*SD* = 15.887). The study utilized a similar methodology, but participants performed only two runs of training (one with real-time feedback) followed by the evaluation run. The pace of the tasks was fixed and slower in comparison to the current study (maximal achievable BTR was = 4 bits/min), consequently producing poor results in terms of BTR (0.373 bits/min).

**Figure 4:**
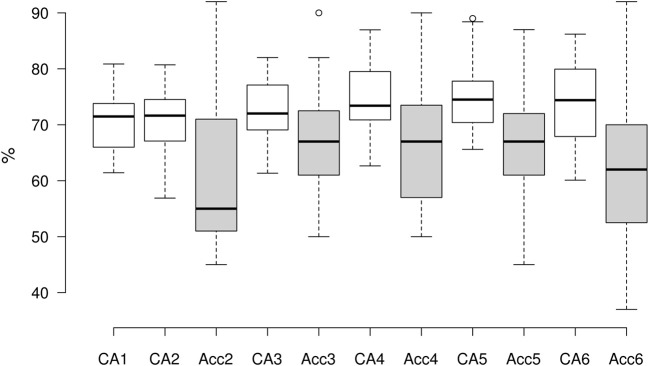
Boxplot showing comparison of on-line accuracy and CA per run side-by-side.

Average best BTR in the current study was 1.992 bits/min (min = 0.280, max = 5.011, *SD* = 1.585), mostly gained in the 6th training run (7 participants), followed by the 5th run (5 participants), 4th run (4 participants) and 3rd run (3 participants). Details on both performance metrics are present in Table 1, for comparison of on-line and classification accuracy per run see Figure 4.

**Table 1:** Average performance (SD in parenthesis) per run and average peak performance.

**Run**	**Accuracy (%)**	**BTR (bits/min)**	**CA (%)**	**ERD (dB)**
1	–	–	70.862 (5.406)	−0.762 (0.443)
2	62.26 (15.566)	0.571 (0.921)	70.115 (6.355)	−1.048 (0.605)
3	67.11 (10.603)	0.825 (0.949)	72.220 (5.441)	−1.117 (0.565)
4	66.74 (10.964)	1.026 (1.227)	74.701 (6.165)	−0.921 (0.595)
5	65.74 (9.683)	1.002 (0.978)	75.081 (6.648)	−0.751 (0.415)
6	62.53 (15.193)	0.992 (1.414)	73.905 (7.651)	−0.550 (0.517)
Best	75.84 (11.251)	1.992 (1.585)	78.991 (4.852)	−1.316 (0.513)

Only one participant was excluded due to poor results (accuracy 47%, 50%, 47% in first three runs), indicating sub-chance on-line accuracy in 1 out of 20 participants.

### 4.2. Classification Accuracy

Grand average classification accuracy (CA) was 72.814% (*SD* = 4.675), while average of peak performance CA in each participant equaled 78.991% (*SD* = 4.852). Both of these values are higher than chance level (65%) for 2-class BCI with 20 trials per class, computed using binomial distribution for a significance level of 5% (Müller-Putz et al., 2008). All participants surpassed the chance level CA in both peak performance and their average session CA.

CA was strongly correlated with the on-line accuracy in all runs of the experiment (*p* < 0.05 in all cases), and the Spearman correlation between average on-line accuracy and average CA was *r* = 0.754, *p* = 0.000 (all correlations in this paper were tested using the Spearman correlation).

### 4.3. ERD Results

From the available mean ERDs per run, two values were calculated; the average ERD per participant and the strongest participant's ERD (“best ERD”). For completeness, we disclose the results of statistical tests for both of these SMR modulation indices where applicable.

All participants were able to produce distinct ERD patterns during the session, while the grand average was a change of -0.858 dB (*SD* = 0.436) from the baseline (more negative ERD value corresponds to a stronger SMR modulation, hence the values of correlations including SMR modulation are negative in case of stronger ERDs associated). The average value of the best ERDs achieved in a run equaled −1.316 dB (*SD* = 0.513).

### 4.4. Embodiment

Median of the SoO statements was 5 (*SD* = 1.190), median SoA 5.5 (*SD* = 0.947). Both of the main embodiment metrics show a positive, moderately strong rating of embodiment. The correlation between these two variables is moderate and statistically non-significant (*r* = 0.357, *p* = 0.134). Due to the lack of a control group in our experiment, it is not possible to measure the increase of embodiment. Nevertheless, our previous study (Škola and Liarokapis, 2018) utilized a very similar embodied virtual training. The main differences between these two studies (with respect to embodiment) are the greater length of the session and elements of gamification added in the current design. This allows us to compare the difference between embodiment variables at least indirectly. Both SoO and SoA were perceived as slightly weaker and with a greater SD in the previous study (SoO mean = 4.7, *SD* = 1.669, SoA mean = 5.4, *SD* = 1.773). It is likely that both the length of the VR training part and added engagement created using the gamification contributed to this increase.

**Figure 5:**
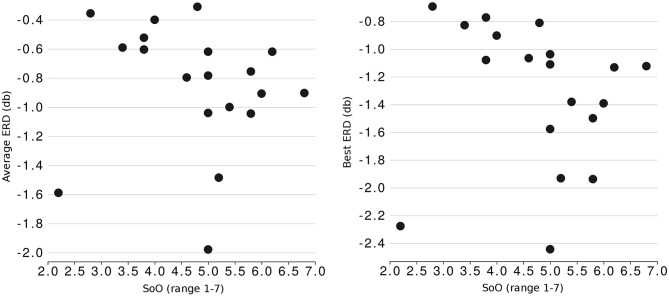
Scatterplot showing the SoO values gathered from questionnaires (x axis) and SMR modulation abilities (y axis). Left plot represents relationship with average ERD across all runs, right plot relationship with best ERD achieved.

Investigation into the relationship between embodiment and the ability to modulate SMR rhythms showed a non-significant correlation between the ownership and the average ERD strength (*r* = −0.371, *p* = 0.118) and a stronger, significant correlation between SoO and the best ERD (*r* = −0.459, *p* = 0.048). Figure 5 showing the correlations between the SoO and ERD reveals the strong linear trend between SMR modulation and the SoO ratings with the exception of an outlying participant #14 (this participant also produced the lowest SoA rating despite being amongst the top performing participants with peak BTR = 3.709). Investigation into this correlation after exclusion of the outlying participant confirmed very strong correlation between the SoO and ERD strength, with an average ERD (*N* = 18, *r* = −0.595, *p* = 0.009) and the best ERD (*N* = 18, *r* = −0.698, *p* = 0.001).

**Figure 6:**
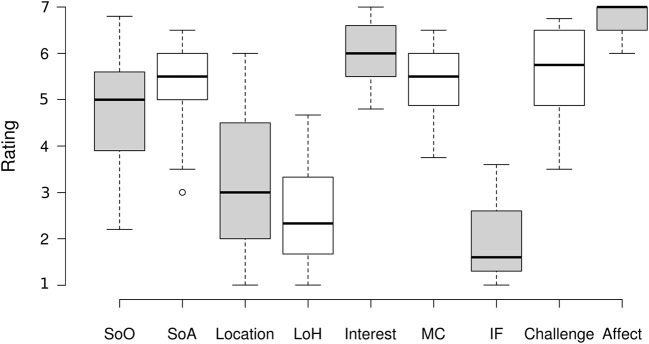
Boxplot with descriptive statistics of questionnaire variables; MC is mastery confidence, IF incompetence fear, and LoH loss of hand.

Interestingly, the correlation between SoA and training results is not present in our data; i.e., no link between participants' perceived performance and their actual performance was established (*r* = 0.002, *p* = 0.994), similarly to our last study on embodied MI-BCI training. Agency measures were correlated (with borderline significance) with two questionnaire scales; the affect (*r* = 0.445, *p* = 0.056) and the embodiment measure of “Loss of Hand” (*r* = 0.445, *p* = 0.056). Descriptive statistics for embodiment are represented in Figure 6.

### 4.5. Motivation and Affect

Affect was linked to better SMR modulation (correlation with average ERD: *r* = −0.436, *p* = 0.062 and with best ERD: *r* = −0.324, *p* = 0.176), but only non-significantly. Statistically significant correlations between pre-experiment questionnaires and performance were revealed only regarding the last training runs. Pre-experiment interest was correlated to BTR in the 6th run (*r* = 0.541, *p* = 0.017) and non-significantly in the 5th run (*r* = 0.399, *p* = 0.091). Similarly, challenge correlated with 6th run BTR (*r* = 0.459, *p* = 0.048) and non-significantly in the 5th run (*r* = 0.430, *p* = 0.066). Challenge was also non-significantly correlated to better SMR modulation in the 6th run (*r* = −0.403, *p* = 0.087). A strong correlation was found also between interest and order of participant's best run in terms of BTR (*r* = 0.559, *p* = 0.013), while a weaker non-significant correlation was present also between challenge and order of best BTR performance (*r* = 0.429, *p* = 0.067). These findings together suggest that participants with greater interest tended to give their best performance in the last runs of the experiment.

Overall, questionnaires revealed a surprisingly high positive affect (median 7, *SD* = 0.348), and no participant reported engagement or interest after the experimental session lower than 6 out of 7 points. See Figure 6 showing descriptive statistics for questionnaire variables.

### 4.6. Other Results

#### 4.6.1. BCI-Naïvity

As both first time users and users with some (although very limited) BCI experience participated in this study, statistical testing was conducted to rule out the effect of previous exposure. Previous experience with MI-BCI did not affect the training results, classification accuracy, or ERDs, in any of the runs (tested using Mann-Whitney *U* test). For clarity, results of the *U*-tests of aggregated results are present in Table 2.

**Table 2:** Aggregated results from *U* tests showing no effect of previous BCI experience in our data.

**Metric**	***U***	***p***
Best CA	51.000	0.661
Avg CA	50.000	0.720
Best accuracy	48.500	0.780
Avg accuracy	44.000	0.968
Best ERD	42.000	0.842
Avg ERD	37.000	0.549
Best BTR	49.000	0.780

Differences were found in fatigue index in the first run (see below) and in the questionnaire scale fear of incompetence (*U* = 19.000, *p* = 0.035), with mean = 2.422 and *SD* = 0.851 in naïve population vs. mean = 1.560 and *SD* = 0.782 in non-naïve.

#### 4.6.2. Gender

It is not possible to draw definite conclusions from the discovered differences between genders, as our data sample is not balanced. However, similar to our previous study (Škola and Liarokapis, 2018), we found females to perform better than males on average.

In terms of on-line accuracy, a statistically significant difference was present in the mean performance; female mean = 70.286%, *SD* = 7.846 and male mean = 61.717%, *SD* = 8.061 (*U* = 68.000, *p* = 0.028).

Females performed better in the 6th run of the experiment (with changed feedback modality), which can be observed as significant differences in terms of both BTR: female mean = 1.896, *SD* = 1.860 and male mean = 0.465, *SD* = 0.748 (*U* = 69.500, *p* = 0.017) and on-line accuracy: female mean = 73.710%, *SD* = 12.880 and male mean = 56.000%, *SD* = 12.692 (*U* = 71.500, *p* = 0.010). Difference in 6th-run SMR modulation is not significant (*U* = 22.000, *p* = 0.100).

Gender had an effect only on the first run CA, female mean = 73.659%, *SD* = 4.864 and male mean = 69.230%, *SD* = 5.199 (*U* = 65.500, *p* = 0.045).

#### 4.6.3. Fatigue

Evolution of the fatigue index per run does not confirm an effect of growing fatigue during the session. The highest fatigue index was present during the 6th run (mean 0.809, *SD* = 2.161), but the values are comparable across other runs: 1st (0.690, *SD* = 0.691), 2nd (0.701, *SD* = 1.632), 3rd (0.405, *SD* = 1.104), 4th (0.597, *SD* = 1.300), and 5th (0.602, *SD* = 1.080). The distribution of the values did not differ significantly (related-samples Friedman's two-way ANOVA χ^2^(5) = 3.271, *p* = 0.658). A curious effect of BCI exposure on fatigue during the first run was found: the BCI-naïve participants had a higher fatigue index (1.062, *SD* = 0.859) than non-naïve (0.355, *SD* = 0.187), with *U* = 13.000, *p* = 0.008. This effect was not replicated in any of the remaining runs.

#### 4.6.4. Qualitative Results

The opportunity to comment on the experiment qualitatively was not used frequently by the participants. One category of comments was concerning the visual appearance of the VR scene; two participants considered the hand movements to appear unnatural, two other comments stated that the visuals indicating asteroid destruction were disturbing (but one of these participants stated this improved eventually). Physical discomfort in the head area was reported by three participants. One participant specifically noted that it would be beneficial to have more time to explore MI strategies.

The rest of the comments were either suggestions to improve the VR environment or BCI tasks with the participants' own ideas, or positive comments regarding the experimental application.

#### 4.6.5. Feedback Modality Change

From the total of 19 participants, 11 participants had worse accuracy in the 6th compared to the previous run and 1 had the same accuracy. The rest of the participants gained better results in the run with delayed feedback compared to the previous run. Wilcoxon signed-rank test did not confirm any significant within-subject differences in trial accuracy between the 6th run and other runs; for the 5th run *Z* = −0.806, *p* = 0.420, for the 4th run *Z* = −1.109, *p* = 0.268.

Examination of SMR modulation indices showed larger differences between the last run and the rest. ERD differences were not significant between the 5th and 6th runs (*Z* = −1.932, *p* = 0.053) and the 1st and 6th runs (*Z* = −1.730, *p* = 0.084), but ERD differed significantly between the 6th run and each of runs 2, 3, and 4 (*p* < 0.01 for all cases). The reader can find the mean and SD values of examined variables per run in Table 1.

## 5. Discussion

This article presented a training environment for MI-BCIs utilizing VR embodiment and gamification with a progressive increase of training pace. Results show the proposed method can effectively train participants for a basic level of MI-BCI operation, comparable to the state-of-the-art (Ahn and Jun, 2015), while steadily increasing BTR in the span of the session. Due to the event-driven training design, the classifier in the core of the BCI system can be re-trained from the annotated datasets at any point in the training procedure.

In our work, we aimed to reinforce the effect of virtual embodiment to facilitate the MI training. This was achieved by designing the VR scene centered around a realistic human-like avatar from the first-person perspective. First-person perspective alone can be a factor strong enough to induce illusions of virtual body ownership (Kokkinara et al., 2016), although more traditionally either synchronization of visuotactile or visuomotor signals is leveraged to produce stronger illusory ownership transfer (see section 2.1 for more details). In this work, visuo-imagery synchrony was employed to induce the embodiment. According to the results, and consistently with previous studies, the SoO illusion was created in the majority of the participants.

Correlations between the ERD strength and questionnaire ratings of the SoO toward avatar's moving hands suggest the subjective level of embodiment is indeed linked to a participant's ability to modulate the SMR. Interestingly, no correlation at all was found between ERDs and SoA statements. Participants tended to self-evaluate the perceived agency as high, regardless of their actual performance. Consequently, the SoA results are not correlated to any of the performance metrics (ERD, accuracy, CA, BTR). The reason why participants' subjective self-evaluation of performance did not reflect their real performance might have its roots in the embodiment and its influence on the SoA. It has been demonstrated that an illusory SoA can be acquired; illusory SoA over walking can be created in seated participants, based only on first-person-perspective virtual embodiment (Kokkinara et al., 2016). This means that participants' ability to assess the level of SoA toward their BCI actions could have been biased by observing the acting avatar embodying them, in turn producing subjective answers uncorrelated with the objective measures.

Additional difficulty arises from the fact that the avatar's movements were used as a proxy for surveying the SoA (see the questionnaires in Supplementary Materials). In our case, the perceived SoA might be in fact composed of two components; the perceived accuracy of the BCI actions, as manifested by the hand movements seen in the VR. Although these two components are in fact perfectly correlated, the agency acquired in the embodied VR experience most likely distorted participants' assessment of their BCI accuracy. Still, such subjective strengthening of the SoA for BCI actions could be beneficial, as increased SoA can facilitate the learning process (it is not uncommon to bias the feedback toward better performance in the early stages of training to support a participant's motivation; Jeunet et al., 2016b).

Our results do not show any correlations between the on-line performance and perceived SoO either. That means that either the used questionnaire does not properly assist investigation of the SoO in the case of MI-BCI-mediated VR embodiment, or that the degree of embodiment does not correlate with the actual performance of the synchronized congruent visuo-imagery trials. The correlation between SoO and SMR modulation ability in our results suggests the former suggestion is likely not valid, so it is probably the relationship between BCI accuracy and the SoO (and also the SoA, as we discussed in the last paragraphs) that needs deeper investigation. Indeed, action- (or agency-) driven illusions are known to arise with bodily movements, but more research is needed to assess the requirements for emergence, strength, and other properties of embodiment mediated by a non-motor, BCI operation. Together with the increased SoA uncorrelated to the actual participant performance, these results suggest that the first-person perspective can contribute to perceived embodiment more than the number or accuracy of correctly performed, self-attributed MI-BCI commands, embodied in avatar's motor actions.

The progressive design influenced the training tasks in each following run; participants had to begin with the recognizable MI faster (otherwise timeout curred), but they did not have to stay in the MI state for as long. Performance results show this approach kept the participants alert and motivated sufficiently to improve their results while it maintained non-increasing levels of EEG correlates of fatigue. It has been shown that the demanding MI process can cause growth of fatigue levels during the training (Talukdar et al., 2019). Growing fatigue can in turn influence the performance by means of losing attention and interest. Our investigation into the influence of fatigue did not confirm increasing levels in the span of the experimental session.

Although the peak performance was not achieved strictly in the last runs of the experiment, SoA and especially the post-experimental affect were very high. This suggests our take on gamification was well-accepted, even though the chosen increase in pace was suboptimal and training pace increase should rather follow individual improvements in the training results in future applications. Games aim for high engagement, and BCI training benefits from high levels of attention, which can be facilitated by engagement. Progress and challenge are common motivational affordances increasing engagement in gamified applications (Hamari et al., 2014). Similarly to overly difficult progression, constant winning leads to loss of engagement. Moreover, in the case of the BCI training, too slow pace wastes the time and cognitive resources of the learner. The goal is to keep the challenge at a reasonable level, which would in turn effectively communicate improvements, leading to an influx of positive emotions connected to winning or achieving a goal (Vorderer et al., 2003).

The last training run in the experimental session leveraged different feedback modality (delayed feedback). Alimardani et al. (2016b) showed that when delays between the MI-BCI command and its outcome were introduced in BCI teleoperation of a robot, participants tolerated larger delays than with motion tracking control. Performance in the last run in our experiment did not differ significantly from the previous runs, despite high variance in the accuracy (comparable to the first run with feedback in the session). Our motivation to incorporate the last run with delayed feedback was to investigate the performance after real-time feedback is removed. In that run, participants relied on the acquired MI skills only, and the evaluation was free from influence of motion observation and vibration feedback.

Vibrations to the corresponding hand were delivered during the periods of MI. Usage of vibrations in MI-BCI feedback is not uncommon (Ahn and Jun, 2012; Leonardis et al., 2012; Yao et al., 2014; Barsotti et al., 2018), and it is known that they can have an influence on the ERD. Indeed, as found in Barsotti et al. (2018), participants in the multimodal feedback group (haptic and visual) produced more stable ERD patterns compared to the visual feedback group. Previous research Lopez et al. (2017) found strengthening of the ERDs in the contralateral motor cortex following focal vibration stimulation. However, the stimulation was delivered for at least 10 min and participants were holding a handgrip during the stimulation and EEG recording. Taken together, there is a body of evidence confirming that the vibrotactile stimulation can help participants to produce more pronounced ERDs during MI, effectively helping them to acquire the BCI operation skills faster. This is especially interesting in case of vibrations producing illusory arm movement sensations (proprioceptive feedback), further helping the embodiment (Leonardis et al., 2012).

### 5.1. Limitations

This study is subject to two main limitations: absence of a control group and a limited length of the experimental intervention. The fact that no control group was employed in the study prevents us from separation of effects found in the results. After comparison of the current data to our previous study that employed a similar design (Škola and Liarokapis, 2018), a clear improvement is shown in the main experimental variables (i.e., BCI performance and virtual embodiment). Nevertheless, based on the single-group results, we cannot argue for specificity of the intervention to the experimental variables, as the comparison of the main variables is indirect, and we lack previous data for comparison in case of motivation and affect. To resolve this problem, further data collection with the aim to form a control group would need to be performed. Correlations found in the data are not affected by this limitation.

The study was performed in the span of one day, limiting the conclusions regarding learning effects. Long-term studies with healthy participants are not very common in the BCI field, and most of the studies are either performing the training in separate sessions over several days (2–4 days are common) or are performed within a single day (see e.g., Leeb et al., 2007; Hwang et al., 2009; Neuper et al., 2009; Alimardani et al., 2013; Sollfrank et al., 2015; Braun et al., 2016). It should be noted that even single-session BCI training can produce large changes in brain signals used for BCI control (Shenoy et al., 2006).

The short length of this study prevents us from making conclusions regarding long-term plastic changes in the brain. Based on our results, we can only demonstrate rapid familiarization to the proposed training procedure and steep increase in accuracy during the first couple of runs, but to prove whether this method can be used to acquire the ability to control BCIs accurately and with consistent performance over longer periods of time, a study spanning days, or preferably weeks would be required.

A limitation of our study that should be mentioned as well is that electromyography was not used to monitor attempted movement during the training and instead, controllers were used to detect movement of the hands. Although this method should cover most of the cases when muscle activity was engaged, it is less rigorous than electromyographical recordings and thus it is possible that participants produced some muscle tension or subtle movements that were not properly discarded from the recording.

## 6. Conclusion

This article presented a gamified VR training for MI-BCIs leveraging body ownership transfer into the avatar to mediate embodied feedback. Contrary to the standard training protocols, our training was designed with the aim to maintain high levels of attention and motivation. This was achieved using a progressively increasing training pace and by providing participants with the information about their progress (using score points). The proposed training method is event-driven, but not externally paced. Event-driven design is especially useful early in the BCI skill acquisition process when classifier training occurs often. This design allows for the creation of datasets for supervised learning while adhering to turn-based game mechanics.

Performance results (on-line accuracy, CA, SMR modulation indices) confirm that the proposed training method improves initial MI-BCI operation skills in first-time and beginner users. Questionnaires indicate very high affect after approximately a half-hour of actual training in the span of a 2-h long session. This, together with low levels of fatigue as assessed from the EEG data, shows that we succeeded in the design of a user-friendly BCI training method. Moreover, pre-experiment motivation and post-experiment affect were both linked to better SMR modulation abilities, further confirming importance of the psychological state of a user in the MI-BCI training process.

We additionally present results concerning the influence of embodiment on MI-BCI control. While the strength of ERDs during session was positively correlated to the subjective magnitude of SoO, the perceived ownership of the avatar body was not correlated to the MI-BCI control performance nor to the SoA. This raises a question of how much the perceived SoA or actual BCI proficiency influence the magnitude of virtual embodiment in case of MI-BCI-induced ownership illusions.

## 7. Future Work

Last run of our experiment showed that removal of the real-time feedback does not necessarily need to negatively affect the performance. Especially participants achieving good results in the last run before the modality change did not have problems with adaptation on the modified training task, often producing the best results in the last run. This fact can be leveraged in design of training environments; training can be more interesting after incorporation of different training tasks, theoretically boosting learning of the MI-BCI skills (suggested in Jeunet et al., 2016b). It enables the design of more complex gamified scenarios that would facilitate the training using variety of training tasks.

Current trend in MI-BCI research is to use a study design that requires participants to be trained with the standard protocol first, and only after they get to a sufficient level of accuracy, the actual research task follows. Results from the last run of our experiment suggest that the skill transfer from the embodied VR training could be done seamlessly after a basic level of performance is achieved. To determine it with confidence, future studies are needed. As the proposed VR training method is comparably effective to the state-of-the-art, using it as a replacement for the standard, abstract-guidance Graz training implementation would likely create more bearable conditions for participants in BCI experiments.

It would be interesting to conduct a deeper investigation into the relationship between the accuracy of the MI-BCI actions and the embodiment during BCI-mediated body ownership transfer illusions. Employment of more detailed, and possibly objective, SoO and SoA measures would further assist investigation of this relationship.

## Data Availability Statement

The raw data supporting the conclusions of this manuscript will be made available by the authors, without undue reservation, to any qualified researcher.

## Ethics Statement

This study was carried out in accordance with the recommendations of Ethics Committee, Masaryk University with written informed consent from all subjects. All subjects gave written informed consent in accordance with the Declaration of Helsinki. The protocol was approved by the Ethics Committee of Masaryk University.

## Author Contributions

FŠ and FL conceived the research. ST designed and implemented the virtual environment for training. FŠ designed the study, performed the data collection, analysis, and wrote the paper. FL and ST provided comments.

### Conflict of Interest

The authors declare that the research was conducted in the absence of any commercial or financial relationships that could be construed as a potential conflict of interest.

## Footnotes

^1^https://www.neuroelectrics.com/products/enobio/

^2^https://www.oculus.com/rift/

^3^https://unity3d.com/unity

^4^https://www.mixamo.com/

^5^Based on information from: https://xinreality.com/wiki/Oculus_Rift
