# Supplementary material for: Progressive Training for Motor Imagery Brain-Computer Interfaces Using Gamification and Virtual Reality Embodiment
Source: Front Hum Neurosci. 2019 Sep 26;13:329. doi: 10.3389/fnhum.2019.00329 (PMC6775193; doi:10.3389/fnhum.2019.00329)
Supplement: Supplementary file 1 [file Data_Sheet_1.PDF]

frontiers

# *Supplementary Material*

This document contains English version of questionnaires used in the experiment. Pre-experiment questionnaire investigated participants' motivation on the following subscales: interest [I], mastery confidence [MC], incompetence fear [IF], and challenge [C]. Post-experiment questionnaire surveyed participant sense of ownership [SoO], sense of agency [SoA], sense of location [Loc], loss of hand [LoH], and affect [Aff].

For purposes of this Supplementary Material, each question was prefixed with indication of the corresponding subscale (e.g., [SoO]). This indication was not present in the questionnaires used in the experiment.

## 1 SUPPLEMENTARY DATA

# Pre-experiment questionnaires

*Please position an 'X' somewhere on the 7-point scale according to the degree of agreement with the following statements.*

*Please, answer the questions truthfully.*

[I] I look forward to work with the BCI (brain-computer interface) today

| | | | | | | |
|---|---|---|---|---|---|---|
| Not at all | | | Somewhat | | | Completely |

[MC] I think I can deal with the difficulties of this task

| | | | | | | |
|---|---|---|---|---|---|---|
| Not at all | | | Somewhat | | | Completely |

[MC] Probably the training will not go well today

| | | | | | | |
|---|---|---|---|---|---|---|
| Not at all | | | Somewhat | | | Completely |

[I] I like improving my strategies or trying out new strategies for the training

| | | | | | | |
|---|---|---|---|---|---|---|
| Not at all | | | Somewhat | | | Completely |

[IF] I feel under pressure to perform well

| | | | | | | |
|---|---|---|---|---|---|---|
| Not at all | | | Somewhat | | | Completely |

[C] The training is a big challenge for me

| | | | | | | |
|---|---|---|---|---|---|---|
| Not at all | | | Somewhat | | | Completely |

[I] I look forward to start with today's training

| | | | | | | |
|---|---|---|---|---|---|---|
| | | | | | | |
| Not at all | | | Somewhat | | | Completely |

[C] I am very curious how I will perform today

| | | | | | | |
|---|---|---|---|---|---|---|
| | | | | | | |
| Not at all | | | Somewhat | | | Completely |

[IF] I dread a little that I can embarrass myself here

| | | | | | | |
|---|---|---|---|---|---|---|
| | | | | | | |
| Not at all | | | Somewhat | | | Completely |

[C] I am fully determined to give my best in the training

| | | | | | | |
|---|---|---|---|---|---|---|
| | | | | | | |
| Not at all | | | Somewhat | | | Completely |

[I] I don't need a reward for the training; I also have fun just like that

| | | | | | | |
|---|---|---|---|---|---|---|
| | | | | | | |
| Not at all | | | Somewhat | | | Completely |

[IF] It's embarrassing for me to fail here

| | | | | | | |
|---|---|---|---|---|---|---|
| | | | | | | |
| Not at all | | | Somewhat | | | Completely |

[MC] I think that everyone can control his/her brain activity

| | | | | | | |
|---|---|---|---|---|---|---|
| | | | | | | |
| Not at all | | | Somewhat | | | Completely |

[MC] I think I won't be able to accomplish the training today

| | | | | | | |
|---|---|---|---|---|---|---|
| | | | | | | |
| Not at all | | | Somewhat | | | Completely |

[C] When I do well in the training today, I will be proud of my achievement

| | | | | | | |
|---|---|---|---|---|---|---|
| | | | | | | |
| Not at all | | | Somewhat | | | Completely |

[IF] I am worried when thinking about the training

| | | | | | | |
|---|---|---|---|---|---|---|
| | | | | | | |
| Not at all | | | Somewhat | | | Completely |

[I] I would also train outside the training hours

| | | | | | | |
|---|---|---|---|---|---|---|
| | | | | | | |
| Not at all | | | Somewhat | | | Completely |

[IF] The training demands paralyze me

| | | | | | | |
|---|---|---|---|---|---|---|
| | | | | | | |
| Not at all | | | Somewhat | | | Completely |

## Post-experiment questionnaires

*Please answer the questions bellow by positioning 'X' somewhere on the 7-point scale.*

During the virtual reality training...

[SoO] ...it seemed like the virtual hands were part of my own body.

| | | | | | | |
|---|---|---|---|---|---|---|
| | | | | | | |
| Not at all | | | Somewhat | | | Completely |

[LoH] ...it seemed like I was unable to move my hands even if I wanted to.

| | | | | | | |
|---|---|---|---|---|---|---|
| | | | | | | |
| Not at all | | | Somewhat | | | Completely |

[SoO] ...it seemed like the virtual hands began to resemble my real hands.

| | | | | | | |
|---|---|---|---|---|---|---|
| | | | | | | |
| Not at all | | | Somewhat | | | Completely |

[SoA] ...it seemed like I was in control of the movements performed by the virtual hands.

| | | | | | | |
|---|---|---|---|---|---|---|
| | | | | | | |
| Not at all | | | Somewhat | | | Completely |

[Loc] ...it seemed like I could feel the movements performed by the virtual hands with my hands.

| | | | | | | |
|---|---|---|---|---|---|---|
| | | | | | | |
| Not at all | | | Somewhat | | | Completely |

[SoO] ...it seemed like I was looking directly at my own hands, rather than at virtual hands.

| | | | | | | |
|---|---|---|---|---|---|---|
| | | | | | | |
| Not at all | | | Somewhat | | | Completely |

[SoA] . . . it seemed like
I was controlling the virtual hand movements by thinking of moving my left or right hand.

| | | | | | | |
|---|---|---|---|---|---|---|

Not at all — Somewhat — Completely

[LoH] . . . it seemed like I couldn't really tell where my hands were.

| | | | | | | |
|---|---|---|---|---|---|---|

Not at all — Somewhat — Completely

[SoO] . . . it seemed like the virtual hands belonged to me.

| | | | | | | |
|---|---|---|---|---|---|---|

Not at all — Somewhat — Completely

[LoH] . . . it seemed like my hands had disappeared.

| | | | | | | |
|---|---|---|---|---|---|---|

Not at all — Somewhat — Completely

[SoO] . . . it seemed like the virtual hands were my hands.

| | | | | | | |
|---|---|---|---|---|---|---|

Not at all — Somewhat — Completely

[Aff] I found that experience interesting.

| | | | | | | |
|---|---|---|---|---|---|---|

Not at all — Somewhat — Completely

[Aff] I found that experience enjoyable.

| | | | | | | |
|---|---|---|---|---|---|---|

Not at all — Somewhat — Completely

## Debriefing Session

*Please use this page to express any comments or thoughts on the experiment:*
